# Identification of the Natural Frequencies of Oscillations of Perforated Vibrosurfaces with Holes of Complex Geometry

**DOI:** 10.3390/ma16175735

**Published:** 2023-08-22

**Authors:** Serhii Kharchenko, Sylwester Samborski, Farida Kharchenko, Andrzej Mitura, Jakub Paśnik, Izabela Korzec

**Affiliations:** 1 Department of Applied Mechanics, Lublin University of Technology, 20-618 Lublin, Poland; 2 Department of Fundamentals of Production Engineering, Lublin University of Technology, 20-618 Lublin, Poland; 3 Department of Agricultural Engineering, Sumy National Agrarian University, 40-021 Sumy, Ukraine; 4 Department of Machine Design and Mechatronics, Lublin University of Technology, 20-618 Lublin, Poland

**Keywords:** finite element method, natural frequencies, vibration forms, visualization, perforated surface, holes of complex geometry

## Abstract

The reliability of perforated vibrosurfaces is one of the main parameters of the efficiency of their operation in many technological processes. Existing methods for studying vibrosurfaces with standard single holes and the corresponding results cannot be used to study the reliability of vibration surfaces with holes of complex geometric shapes. The proposed method is based on the experimental modal identification of the parameters of natural oscillations, the parallel creation of a numerical model using the finite element method, and the comparison of the results. Three vibrosurfaces were investigated: solid without holes, perforated with standard round holes, perforated with holes in the form of a five-petal epicycloid. As a result of experiments, the divergence of natural vibrations of perforated surfaces depending on the side of the punch and matrix during their technological production by pressing was established. The result of the research was a refined adequate numerical model that takes into account the presence of holes in complex geometric shapes. A methodology has been developed, and analytical expressions with perforation coefficients have been obtained, which allow obtaining values of natural oscillations of vibration surfaces depending on the properties of metal, boundary conditions, and structural and kinematic parameters.

## 1. Introduction

The use of perforated surfaces as working elements of machines for the agricultural, chemical, food, construction, and mining industries, in most cases, involves the presence of vibration, which affects their reliability [1,2,3,4,5,6].

The promising use of perforations with holes of complex geometry proved a significant intensification of productivity and quality of technological indicators, despite the lack of reliability studies [7].

The use of holes with complex geometric shapes has proven promising in terms of productivity and quality of technological processes [8]. Thus, the use of five-petal epicycloid shape holes showed an increase in productivity by 80–100% compared to the basic round holes in the separation of grain mixtures of peas and chickpeas [9]. 

However, in the literature, there is no methodology for studying the natural frequencies and vibration shapes of such perforated surfaces and the corresponding dependencies.

When studying the design of a perforated surface, it is important to know its natural frequencies, which are subject to various excitations. Different methods are used to study the vibrations of plates: theoretical (analytical), numerical, and experimental [4,10,11,12,13,14,15,16,17,18].

The application of the analytical method of analysis, in most cases, is focused on the study of variable boundary conditions, isotropic and orthotropic properties, and the single arrangement of holes [19].

The effect of the presence of a single hole of various shapes on the free oscillations of a rectangular plate has been studied by applying a discrete solution [20], the ICCM method [21], and the Rayleigh–Ritz method [22].

Numerical methods are widely used in the study of perforated plates due to their sufficient accuracy and minimal labour input [23,24]. However, the unilateral use of numerical methods, without the use of known results or additional clarifying experiments, can lead to a significant decrease in the accuracy of the results.

A synthesis of different methods makes it possible to obtain the most accurate results in the study of plate oscillations. Thus, studies of a rectangular plate, which is made of various materials and has variable boundary conditions, carried out by experimental and numerical methods, made it possible to obtain adequate results [25,26,27].

A review of the literature shows that most studies focus on plates with single holes of various shapes and given boundary conditions [28,29]. Both analytical and experimental methods are used. The presence of a set of holes on the plate, which have a complex geometric shape, complicates the analytical methods of its analysis. The use of modal and numerical analysis for such structures is the most appropriate for studying the natural frequency of vibrations.

Modal analysis is widely used as a means of experimentally solving engineering problems related to vibration [30]. It allows us to experimentally determine the modal parameters of the studied structure, in particular, natural frequencies, damping coefficients, and eigenforms of oscillations.

Perforated vibration surfaces with basic round holes, which are the most common, and vibration surfaces with holes of complex geometry in the form of a five-petal epicycloid were chosen for the study [9]. The identification of the natural frequencies of oscillations of such surfaces involved the use of a complex methodology, which is based on the use of numerical simulation modeling (FEM) and experimental modal analysis.

The choice of sieves with holes in the form of a five-petal epicycloid is justified by the following. Intensification of the sifting process of loose medium particles through such holes in comparison with holes of regular geometrical shapes is proved. The studied perforated plate has many holes in the form of a five-petal epicycloid, which consists of curved sections. This does not allow or significantly limit the use of standard techniques. The difference of the developed technique is an integrated approach of experimental, numerical methods, with a comparative analysis of the basic structures of the plates (solid non-perforated, with basic round holes). The result of the developed technique was also analytical expressions for identifying the natural oscillation frequencies of plates with holes of complex geometry.

The research was based on experimental and numerical methods for studying oscillations of perforated surfaces, which involved experimental determination of the natural frequencies of the prototype on laboratory equipment and simulation modeling of the research object using finite element methods.

Simcenter Testlab software was used to experimentally determine the natural frequencies of oscillations, which allowed experimental testing and simulation to be combined during the development, design, manufacture, testing, and trials of technical products. Simcenter Testlab software combines reliable data acquisition with high-performance analysis and final reports.

The purpose of modal analysis is to determine the natural frequencies, modal damping, and structural vibration shapes obtained from measured data. The algorithms of Simcenter Testlab software allow the identification of modal parameters to obtain accurate modal estimates from the frequency response functions or operational data.

## 2. Research Methodology

To determine the reliability of perforated surfaces with holes of complex geometry (HCG), the study was divided into stages: modal analysis in the form of an experimental determination of the frequency response of the prototype on laboratory equipment, simulation modeling of the research object using finite element methods (FEM) with ABAQUS software, generalisation of experimental and simulation results, and the definition of finite models for the reliability of perforated surfaces.

During a modal impact test, the frequency response function is established to determine the natural frequencies of the test surface. In a physical sense, the frequency response function is a measure of the system’s output in response (usually acceleration, velocity, or displacement) to a known input (usually force).

The experimental determination of the structural function of the frequency response consists in identifying the natural frequencies of oscillation of the test surfaces, for which the laboratory equipment was developed, and the following methodology was used.

**Figure 1:**
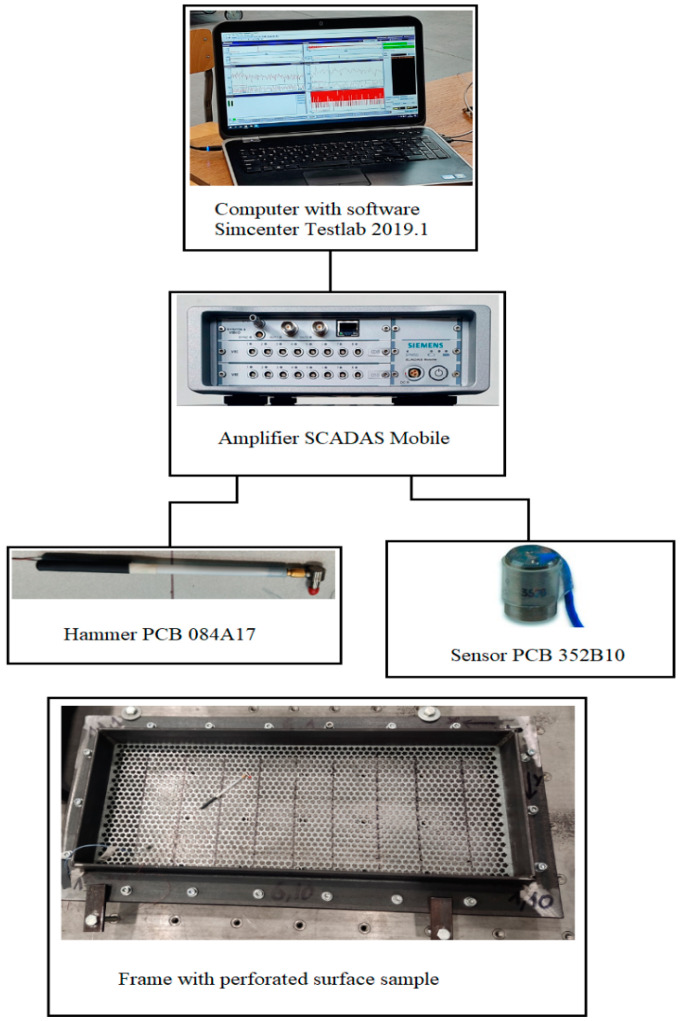
Scheme of equipment for experimental research.

To determine the structural function of the frequency response, it is necessary to obtain two data channels: the input force and the corresponding response of the test object (test surface). In impact measurement, the input force is provided by a modal impact hammer, and the output response of the test object (test surface) is measured using an accelerometer (Figure 1).

The corresponding studies scheme of experimental determination of the natural oscillations of the perforated surfaces prototypes is shown in Figure 1.

The basic elements of the scheme are a special impact pulse type hammer PCB 084A17 for creating excitations (oscillations); cables for signals transmission; accelerometer sensor PCB 352V10 with highly sensitive piezoelectric elements for fixing oscillations; signal amplifier SIEMENS model SCADAS Mobile; computer with Simcenter Testlab 2019.1 software for processing and visualization test results.

The study was conducted according to the following algorithms:

1. Test setup: boundary conditions; determination of test scheme and parameters; frequency range; determination of excitation source and force level.
2. Testing: installation and control of accelerometers; object excitation and frequency response measurement; check of measurement quality and coherence.
3. Post-test: modal curve fitting; validation of the modality against the assurance criterion and modal synthesis.

The research was carried out using the following algorithm. The perforated surface prototype was rigidly fixed to the prefabricated frame. With this type of fixation, the investigated surface at the periphery is fixed and unable to move.

**Figure 2:**
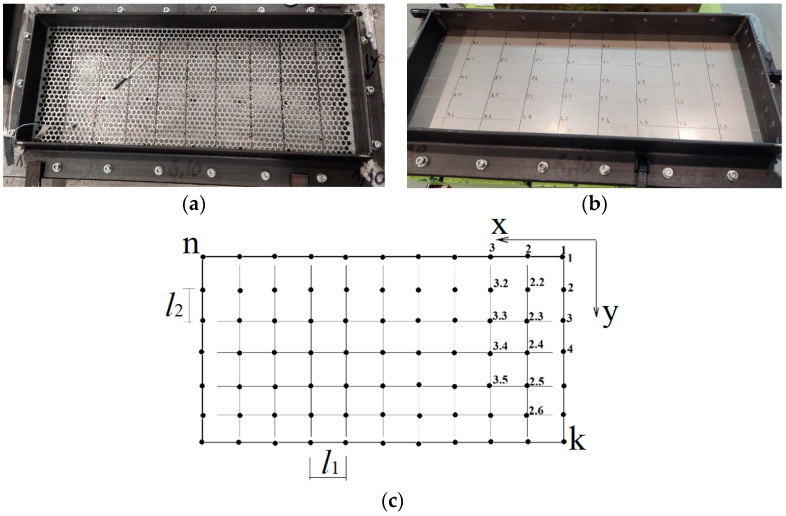
Partitioning the coordinate grid on the prototypes: (**a**)—perforated surface with five-petal epicycloid holes; (**b**)—solid non-perforated metal sheet; (**c**)—scheme of measurement points placement.

The surface of the prototype was marked by overlaying a coordinate grid with the specified step (Figure 2). Marked measuring points and coordinate axes.

A series of points on the test surface form the geometric layout, which will be analyzed. The designation of measurement points is accepted in accordance with axes (*x*,*y*). The direction (±*x*,*y*,*z*) for the measuring channel has been introduced for the correct animation of the modal characteristics. Each point was determined by the coordinate of location on the plane by coordinates (*l*_1_, *l*_2_) (Figure 2c, Table 1).

The PCB 352B10 sensor was glued to the surface of the prototype and connected to the SCADAS Mobile amplifier.

An electrodynamic vibrator or impact hammer was used for excitation in laboratory modal tests. The vibrator allows precise force control but requires careful positioning for its application and increased installation time. With a significant number of measurement points, several vibrators are used. The impact hammer does not require additional adjustment or special installation; however, it creates a variable force. The variation in force is leveled by the repetition of experiments. For an adequate measurement, the input force must: excite a wide frequency range with a high amplitude (above the noise level of the equipment); have an amplitude that is evenly distributed over the frequency.

**Figure 3:**
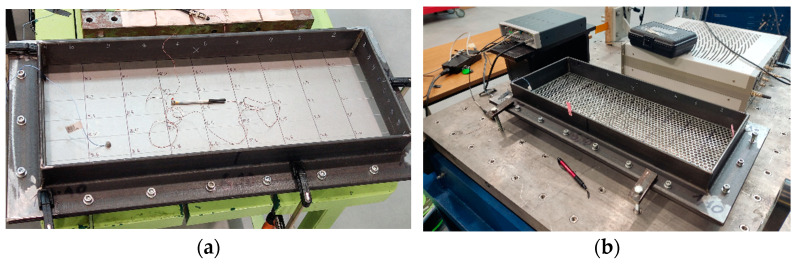
Excitation with a hammer at defined points on the prototypes: (**a**)—solid non-perforated metal sheet; (**b**)—perforated surface with five-petal epicycloid holes.

The modal hammer model PCB 084A17, which is also connected to the SCADAS Mobile amplifier, was used to generate pulse excitation on the test surfaces. The hammer makes it possible to quickly affect multiple points without changing the sensor configuration. In addition, the hammer design provides access to places that are difficult to reach (in our case, the points are located near the attachment frame). The hammer was used to strike the points of a given coordinate grid on the prototype surface (Figure 3).

The number of strokes at each point was five repetitions. Modal testing using a hammer is an ideal solution for studying perforated surfaces, taking into account the availability, minimization of equipment, accuracy, and adequacy of experiments.

The modal parameters of perforated surfaces can be predicted using mathematical models constructed by finite element analysis. Such a model consists of discrete points interconnected by elements whose mathematical properties correspond to the characteristics of the materials of the structure. Boundary conditions are introduced into the model, which determines the method of fastening the structure to the base or its location on the supports, as well as the loads applied to it. A mathematical algorithm is applied to the constructed model, by means of which the eigenforms and frequencies of oscillations are determined.

The finite element method (FEM) was also used for research based on the Abaqus software/CAE 2020.

Once the design is performed, it is helpful to check it. Comparing the results of this analysis with theoretical predictions from the finite-element model allows us to identify and correct errors in the model.

The next step was to compare the results of the finite element model and the results obtained by experimental modal analysis. At the same time, the definition of modeling error was carried out.

With a discrepancy not exceeding 5%, the finite element model was further used to determine the regularities of changes in the natural frequencies of surfaces depending on their complex relative parameters, the absolute parameters of holes and partitions, and the properties of materials.

## 3. Equipment and Materials

The following equipment and materials were used for research:

### 3.1. Types of Investigated Surfaces

**Figure 4:**
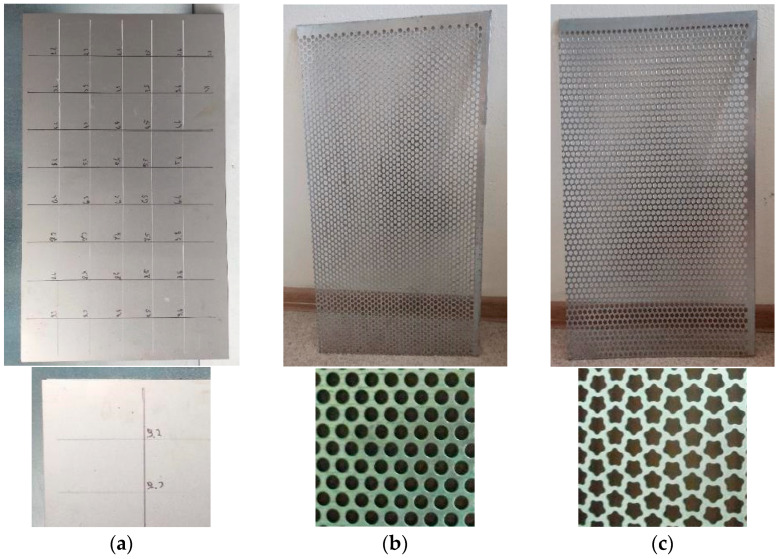
Surface samples during studies: (**a**)—solid sheet; (**b**)—perforated sieve with basic round holes; (**c**)—perforated sieve with holes of complex geometry in the shape of five-petal epicycloid.

Three main types have been selected for research: solid sheet without perforations (Figure 4a); perforated sieve with basic round holes (Figure 4b); perforated sieve with holes of complex geometry in the shape of five-petal epicycloid (Figure 4c).

The test surfaces are made from steel S235 JR according to the EN 10025-2 standard with the corresponding characteristics (Table 2).

### 3.2. The Hammer

**Figure 5:**
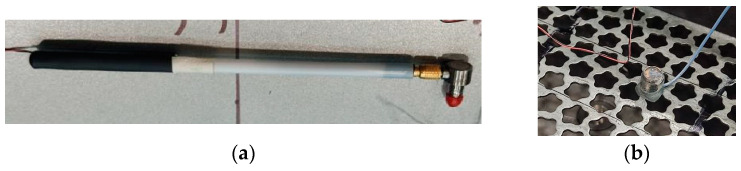
General view of the hammer (**a**) and accelerometer sensor (**b**).

The Hammer (Figure 5a) has the following main characteristics: model PCB 084A17; sensitivity 20 millivolt/newton (mV/N), pulse type.

### 3.3. The Pulse-Type Sensor

The Pulse-Type Sensor (Figure 5b, Table 3) has the following main characteristics: model PCB 352B10; sensitivity 10.31 millivolt/newton (mV/N) [31].

The PCB Piezotronics miniature accelerometers feature highly sensitive piezoceramic elements that operate in a shear scheme and have an integral ICP preamplifier.

The advantages of these accelerometers include the necessary signal-to-noise ratio, high resolution during measurements, and the possibility of studies with the presence of vibrations. The sensitivity of the ceramic ICP preamplifier element determines the minimum weight, wide frequency range, and low noise level compared to a similar quartz module.

The accelerometer’s built-in microelectronic preamplifier is powered by a direct current source. The sensor’s power supply voltage and output signal are transmitted simultaneously over two wires.

**Figure 6:**
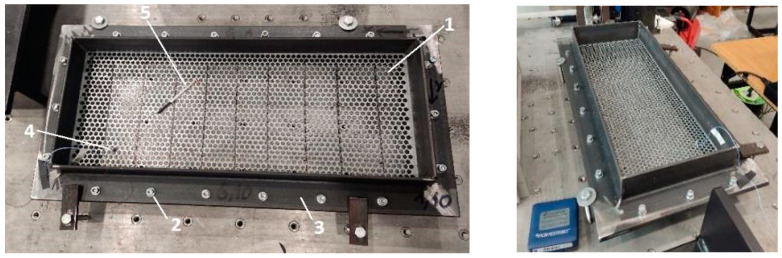
Installation of the sieve in the research frame: 1—sieve; 2—bolted joints; 3—fixed frame; 4—sensor; 5—hammer.

The experience of using experimental modal analysis (beams, plates, etc.) made it possible to provide conditions under which sufficient measurement accuracy was obtained. Thus, to minimize the influence of the sensor weight, the following conditions were adopted: the sensor with the minimum mass in relation to the weight of the plates under study (Figure 4); the coordinates of the sensor location (Figure 6) relative to the frame-restrictor (adjacent zone) and the nodal lines of the modes (forms) of vibrations under study (removal).

The sensor weight ratio (0.7g) to the test plate weight is low. For example, for a plate without perforation (Figure 4a), the ratio is 0.7g/1565g = 0.00044(-) = 0.044%; for the perforated plate with basic round holes: 0.7g/934g = 0.0007(-) = 0.07%; for the perforated plate with holes of complex geometry: 0.7g/823g = 0.0008(-) = 0.08%. Also, the accepted conditions regarding the position of the sensor were the absence of coincidence with the points at which the amplitudes of the modes oscillations will have extremes. The position of the sensor is distant from the nodal lines of the studied modes of oscillations. Thus, based on these assumptions, the mass of the sensor was excluded from the subsequent analysis by the finite element method.

### 3.4. Frame for Fixing Prototypes

The frame (Figure 6) has been developed for the research, which allows the sieve to be fixed rigidly around the periphery and ensures its immovability. Bolted joints and clamps are used for this purpose.

### 3.5. SIEMENS Amplifier SCADAS

Mobile hardware is designed for testing productivity and covers a wide range of noise, vibration, durability, and multi-physics applications.

The device integrates with a special and adapted software package for accelerated measurement configuration and correct formatting of Simcenter Testlab results and analysis. SCADAS Mobile technical specifications [32]: up to 204.8 kHz sampling rate per channel and throughput up to 14 MSamples/s; 24-bit delta-sigma ADC technology; 150-dB dynamic range; can include integrated CAN bus, dual tachometer, and signal generator support; master–slave configurations for distributed systems and channel expansion; high-speed Ethernet host interface; standard (MIL-STD)-810F qualified for shock and vibration.

### 3.6. Software

**Figure 7:**
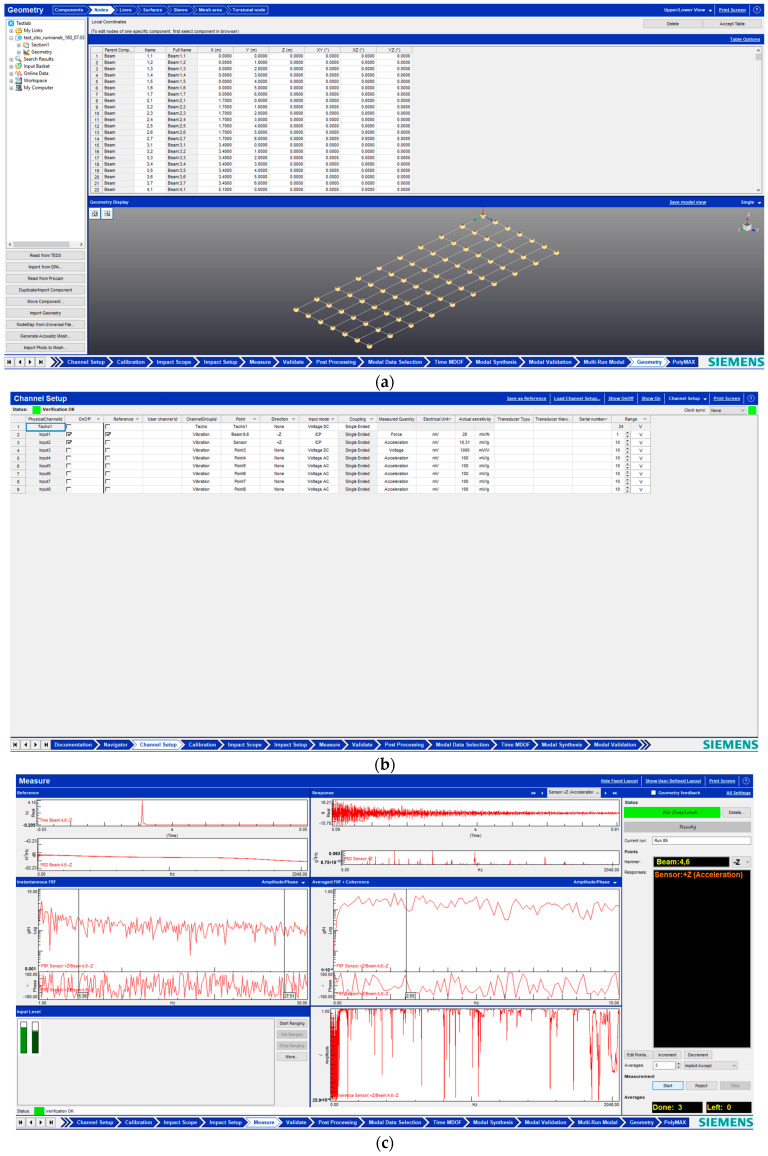
Data processing and visualization stages: (**a**)—entering coordinates of measurement points and creation of geometric layout; (**b**)—adjustment of hammer and accelerometer channels; (**c**)—conducting an experiment at a given point on the surface.

**Figure 8:**
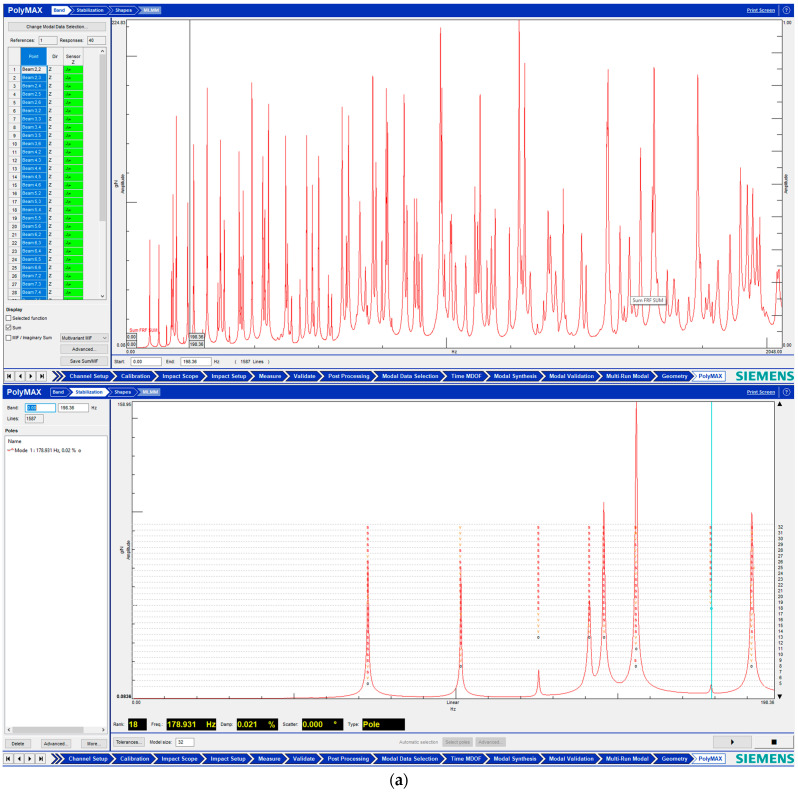

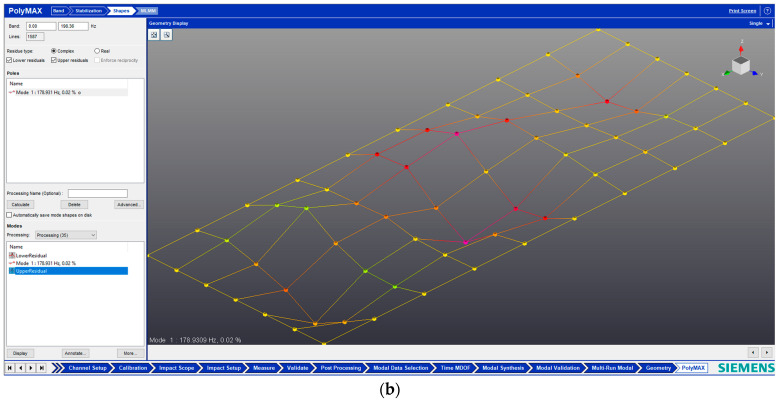
Data processing and visualization stages: (**a**)—processing results, modal curve fitting; (**b**)—visualization of results.

Simcenter Testlab 2019.1 software was used for experimental modal analysis, Abaqus/CAE 2020—finite element model. This software works on the principle of Model-based Development (MBD) and involves the use of simulation modeling and a significant reduction in the number of physical tests. Data processing, results, and visualization are presented in Figure 7 and Figure 8.

**Figure 9:**
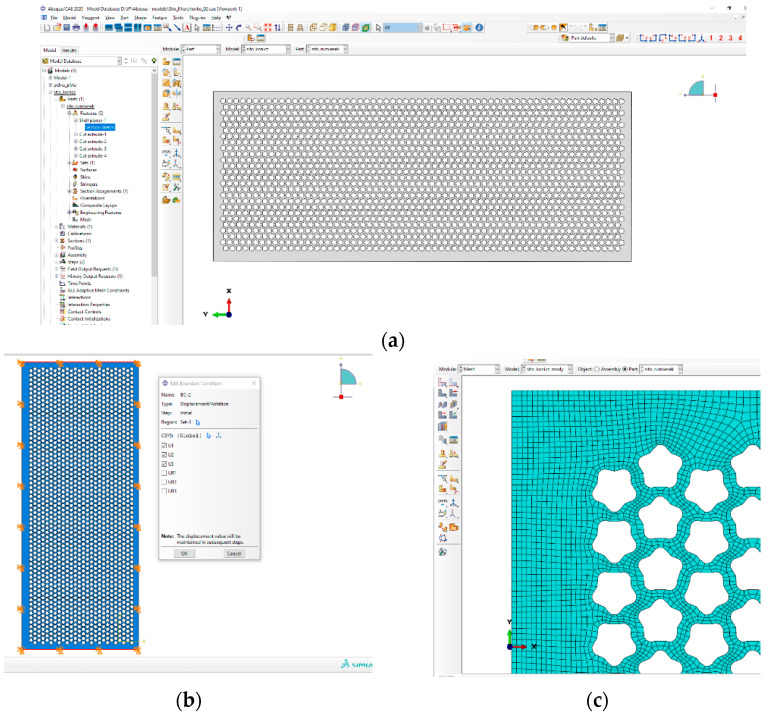

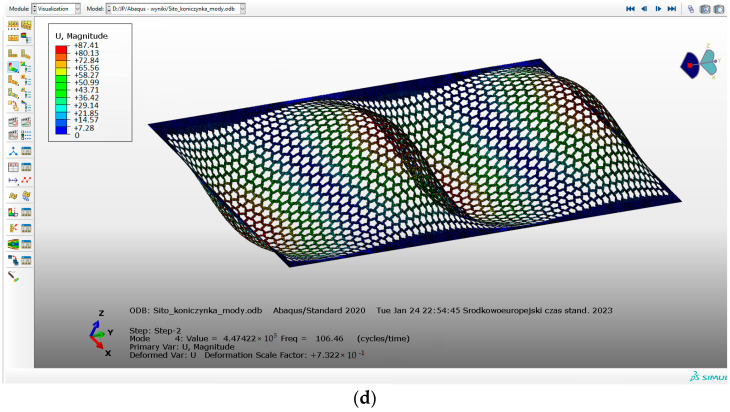
Fragments of research stages in Abaqus/CAE 2020: (**a**)—creating geometry (PART module); (**b**)—setting initial and boundary conditions in displacements (LOAD module); (**c**)—input of element type and parameters (MESH module); (**d**)—visualization of results (VIZUALIZATION module).

Abaqus software was used to build the finite element model (Figure 9).

The main stages of modeling a task in Abaqus (Figure 9): Creating a geometric model → Setting the material properties and parameters → Setting the boundary conditions → Constructing a finite element grid → Running the calculation, analysis, and visualization of the results.

The investigated vibrosurfaces have a rectangular shape and are naturally divided into rectangles, so initially, the quadrangular shell elements of the first-order S4R were used for modeling.

However, as a comparison with the preliminary experiment showed, the use of S4R does not allow to determine the values of surface oscillations with sufficient accuracy. This is due to the complex geometry of the holes with small radii of curvature (compared to the dimensions of the vibration surface). For example, the presence of a given radius of curvature element of epicycloid shape hole with the radius of the inscribed circle (R = 3.5 mm) with overall dimensions of the vibration surface of 640 × 250 mm and a thickness of 1.0 mm. Preliminary testing of the application of different types of elements in Abaqus. In addition, S4R elements were tested: S3R, continuum-shells elements SC6R and SC8R, as well as volumetric elements C3D8R and C3D6.

It is established that the use of continuum-shells and integral elements leads to a significant increase in the calculation time because the calculation time is inversely dependent on the characteristic size of the elements, and for these elements, the characteristic size is the thickness. In addition, when using three-dimensional elements, their number should be significantly increased compared to shell elements. From a comparison of the elements S4R and S3R, it turned out that the results of surface oscillations in triangular elements are more adequate before experiments than in quadrangular ones. Therefore, it was subsequently decided to use S3R elements with an improved grid where necessary.

The following conditions and parameters were adopted for numerical modeling in Abaqus software: the linear model of elastic material, material characteristics in the form of density, Young’s modulus, and Poisson’s ratio (Table 2); finite element mesh: shell elements (S4—without reduced integration); the size of the final element—1 mm; the total number of model elements—166573.

### 3.7. Fractography of Perforated Surfaces

The industrial production of perforated surfaces is predominantly based on the cold pressing of sheet metal. In this case, the work tools used are matrices and punches. The process of extruding a hole produces some geometric variations in the edges of the holes.

**Figure 10:**
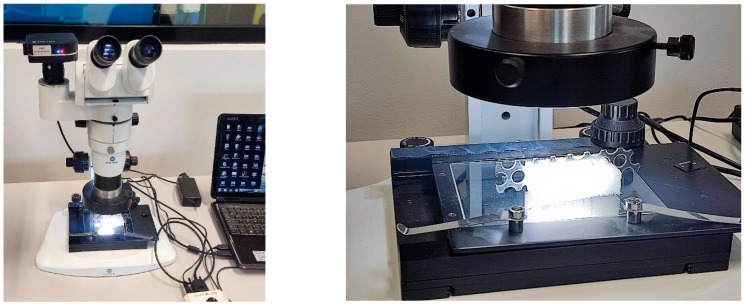
Fractography of the edges of perforated surfaces.

To study the geometric deviations in the perforation edges and their further influence on the natural oscillations of the perforated surfaces, their fractography has been carried out [33,34,35]. Opta-tech × 2000 microscope was used for research (Figure 10). Main characteristics: zoom 1:10/0.8×–8×; equipped with a clik-stop mechanism; adjustable eyepiece distance between 45–76 mm; planachromatic lens; field of view 10 × 22 mm; EPI/DIA illuminator brightness adjustment; LED lighting.

**Figure 11:**
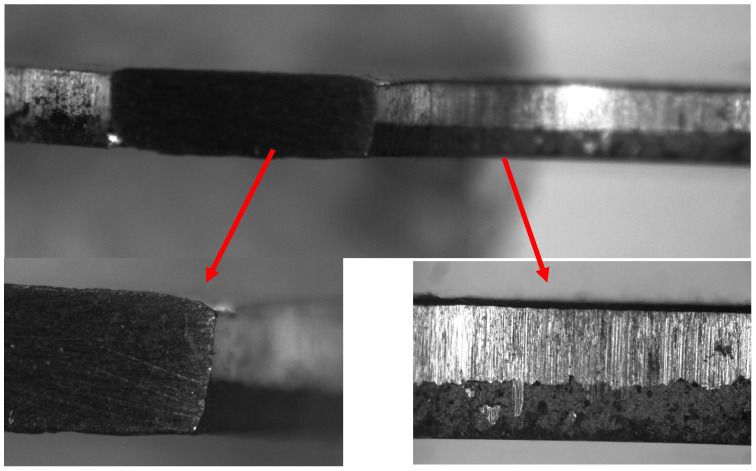

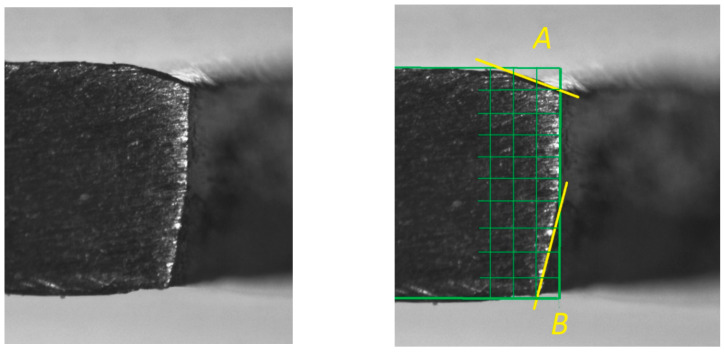
Edge images of perforated surfaces.

The results of the studies are shown in Figure 11.

Analysis of the obtained images established atypical geometric deviations: zones A and B (Figure 11). It should be noted that zone A is on the punch side, and zone B is on the reverse side of the matrix.

For convenience, a grid with a pitch of 0.1 mm is applied, which identifies the corresponding zones A and B. Zone A determines the bevel of the edge with dimensions of 0.11 × 0.25 mm, zone B—0.1 × 0.38 mm.

For ease of further study, we introduce relative parameters with respect to the thickness (*h*) of the surface and the radius of the holes (R):

Δ_AX_ = (*l*_AX_/R) 100%; Δ_AY_ = (*l*_AY_/*h*) 100%; Δ_BX_ = (*l*_BX_/R) 100%; Δ_BX_ = (*l*_BY_/*h*) 100%, (1)

where *l*_AX_, *l*_AY_—the bevel length of zone A along the *x*-axis and *y*-axis, respectively; *l*_BX_, *l*_BY_—the bevel length of zone B along the *x*-axis and *y*-axis, respectively.

The obtained results of calculations are entered in Table 4.

The study of the influence of such geometric deviations of the edges of perforated surfaces on their frequency of oscillations was also included in the tasks of these studies.

In the course of the research, technological deviations were found in the geometry of the hole edges during their manufacture by mechanical stamping. Their quantitative absolute and relative parameters are established (Figure 11, Table 4). The effect of these deviations on the oscillation frequency of perforated plates has been experimentally established. In addition, the lack of consideration of these geometric deviations of the various hole edges in the numerical simulation of FEM leads to a decrease in the accuracy of calculations. Based on the results of the research, it is recommended to take into account these edge deviations when modeling by numerical methods at the stage of constructing the geometry of the plate.

## 4. Results

The most common types of oscillations were selected for the study (Table 5). Eight common modes (forms of oscillations), which are typical for separating sieve machines and extending the area of further practical application, have been selected for research. These forms of oscillations for a given design of perforated vibrating plates provide a certain range of variation in the amplitude of oscillations. The range of oscillation amplitude variation was: for one moda (Table 5) U = 0–9.58 × 10^1^ mm, two moda—U = 0–1.037 × 10^2^ mm, three moda—U = 0–1.015 × 10^2^ mm, four moda—U = 0–9.541 × 10^1^ mm, five moda—U = 0–9.989 × 10^1^ mm, six moda—U = 0–9.434 × 10^1^ mm, seven moda—U = 0–9.408 × 10^1^ mm, eight moda—U = 0–9.394 × 10^1^ mm.

As a result of the research, we will reduce the resonant peaks at each frequency.

### 4.1. Study of the Natural Frequency of Oscillations of a Solid Plate

According to the proposed methodology, a continuous (without holes) metal surface was investigated first (Figure 4a). This surface is a reference (starting point) for the study of surfaces with varying degrees of perforation.

The natural frequency of a continuous surface was studied by experimental (modal analysis), analytical, and numerical (FEM) methods.

For the experimental study, modal analysis on Simcenter was used, and for numerical calculation, the Abaqus software environment was used. For calculations by the analytical method, note the Cartesian coordinate system *xyz*, according to which a rectangular plate has length *a* (o*x*), width *b* (oy), and thickness *h* (o*z*).

The equation of oscillations within the Kirchhoff–Lyava hypotheses has the form [36]:

(2) $$D\Delta \Delta \omega +\rho h\frac{\partial^{2}\omega }{\partial t^{2}}=q,$$

where *ω*—plate deflection; $D=\frac{Eh^{3}}{12(1-\mu^{2})}$—cylindrical stiffness; *ρ*—material density; *q*(*x*,*y*,*t*)—transverse load intensity; *E*—modulus of elasticity; *μ*—Poisson’s ratio; $\Delta =\frac{1}{H_{1}H_{2}}\left[\frac{\partial }{\partial x}\left(\frac{H_{2}}{H_{1}}\frac{\partial }{\partial x}\right)+\frac{\partial }{\partial y}\left(\frac{H_{1}}{H_{2}}\frac{\partial }{\partial y}\right)\right]$—generalized Laplace operator; *H*_1_, *H*_2_—Lamé parameters.

Under the accepted boundary conditions, when *H*_1_ = *H*_2_ = 1, we have

(3) $$\Delta =\frac{\partial^{2}}{\partial x^{2}}+\frac{\partial^{2}}{\partial y^{2}}.$$

The natural oscillations of a rectangular plate with sides *a* and *b* are determined from the expression:

(4) $$\omega =\omega_{0}\sin\frac{\pi m}{a}x\sin\frac{\pi n}{b}y,$$

where *m*, *n*—number of half-waves in the direction of axes *x* and *y*, respectively (*m*, *n* = 1, 2, 3…).

Expression (2) satisfies boundary conditions, which for the edge *x* = 0, *a* have the form:

(5) $$\omega =0,M_{x}=-D\left(\frac{\partial^{2}\omega }{\partial x_{}^{2}}+v\frac{\partial^{2}\omega }{\partial y_{}^{2}}\right)=0.$$

This condition leads to $\omega =\frac{\partial^{2}\omega }{\partial y_{}^{2}}=0$ at *x* = *a*.

Then the second condition $\frac{\partial^{2}\omega }{\partial x_{}^{2}}=0$.

Then, substituting the expression (4) in (2), taking into account (3), we have

(6) $$\omega =\pi^{2}\left(\frac{m^{2}}{a^{2}}+\frac{n^{2}}{b^{2}}\right)\sqrt{\frac{D}{\rho h}}.$$

The use of Edman’s method [36,37,38] made it possible to convert (6) into the expression for determining the natural oscillations of a rectangular plate restrained along the contour:

(7) $$\omega =\frac{\alpha }{a^{2}}\sqrt{\frac{D}{\rho h}},$$

where *α*—frequency factor that takes into account the number of half-waves (*m*, *n*) and the aspect ratio of the plate (*a*/*b*) [36,39].

It should be noted that in [37], the calculations were carried out for a limited ratio *a*/*b* = 1.0; 1.5; 2. The application of the asymptotic method for determining the natural frequency of oscillations of a pinched rectangular plate at higher aspect ratios (*a*/*b* = 2; 4) was carried out in [40].

Taking into account the data of the work [38]: *E* = 2.1 × 10^11^ Pa, *h* = 0.001 m, *μ* = 0.3, *a* = 0.7m, *ρ* = 7847 kg/m^3^, we obtain the values of the oscillation coefficient for various conditions (Table 6).

The presence of perforation in a metal plate can be modeled by introducing a correction factor (*K_p_*) into (7), which generally takes into account the design parameters of the holes, their number, and their location on the plate:

(8) $$\omega =\frac{K_{p}\alpha }{a^{2}}\sqrt{\frac{D}{\rho h}}.$$

Then, the value of *K_p_* = 1 will be for a solid surface. The practice of using such coefficients can be seen in [41], where the author took into account the effect on the natural oscillations of the presence of a single notch (hole) in the plate. The methodology involved the use of a coefficient taking into account the size, shape, and location of the notch and showed sufficient accuracy of the results.

Determination of the natural frequency of plate vibrations with different parameters of perforation, further comparing them with the values of vibrations of a solid (without holes) plate, will allow us to determine with sufficient accuracy the values of the coefficient *K_p_*.

Analysis of the values of the natural frequency of oscillations obtained by experimental (*ω_ES_*), analytical (*ω_TS_*), and numerical (*ω_FS_*) methods showed sufficient accuracy in the presence of a deviation of up to 5% (Table 6).

### 4.2. Study of the Natural Frequency of Oscillations of Sieves with Round Holes

The second stage of the research was the study of the natural frequency of oscillations of sieves with perforations of standard round holes (Figure 4b).

The methodology involved experiments in the form of modal analysis and numerical FEM studies. The obtained results were compared with the results of vibrations of a solid metal plate.

In addition, the tasks of the research included the study of the influence of the technological consequences of the industrial serial production of perforated surfaces by cold stamping on the natural frequency of oscillations. The corresponding consequences can be divided into two types: the deviations of edge angles (Figure 11) and the presence of curvature on the perforated surface.

For this purpose, the examined surface was studied from two sides: from the side of the punch action (0°) and from the side of the matrix action (180°) (during manufacture).

The results of experiments and numerical calculations on FEM are shown in Table 7.

The results of the experimental study of the surface’s natural oscillations are reliable and accepted as a basis. Then, the deviation between the results of the FEM technique and the experiment can be determined by the expression:

*δ* = (*ω_F_* − *ω_E_*/*ω_F_*) 100%, (9)

where *ω_F_*—frequency obtained by the methodology FEM; *ω_E_*—frequency obtained experimentally.

The highest deviation in the values of the natural frequency of oscillations obtained experimentally and by FE-modelling was obtained at 1, 3, and 5 modes and amounted to *δ_R_*_1,2_ = 4.04–10.1% (Table 7), where the transverse axes of oscillations are characteristic (Table 5). The deviation of the natural frequency of oscillations for other modes (2, 4, 6–8) is in the range *δ_R_*_1,2_ = 0.49–4.73% (Table 7). This can be explained by the overall dimensions and rectangular shape of the investigated samples.

As a result of experimental studies, the effect of technological consequences during the manufacture of the perforated surface on its natural frequency of oscillation, which differs by 4.52–12.8% when rotated by 180 degrees (Table 8), was established.

A similar fact explains the deviations of the values of the natural frequency of oscillations obtained experimentally and with the help of FEM. The creation of geometry in FEM in the study of perforated surfaces requires taking into account the corresponding technological consequences in the form of geometric deviations of the edges (Figure 11).

Another possible way to account for this phenomenon is to average the values of the natural frequency of oscillations of each side of the perforated surface in the form of arithmetic mean ω_ERA_ (Table 7). Then, we have an increase in the accuracy of identification to δ_RA_ = 0.24–3%, which is sufficient for further research or engineering design.

### 4.3. Investigation of the Natural Frequency of Oscillations of Sieves with Holes of Complex Geometry

The third stage of research was the study of the natural frequency of oscillations of sieves with holes of complex geometry in the form of a five-petal epicycloid (Figure 4c). The research methodology was similar to Section 4.2. The results of experiments and numerical calculations on FEM are shown in Table 9 and Table 10.

Analysis of the results (Table 9 and Table 10) established a similar effect of the technological consequences of the manufacture of sieves on their natural frequency of oscillations. As for the sieves with round holes, a significant deviation of the natural frequency of oscillations was found *δ_EG_* = 13.61–17.1% (Table 10) experimentally at 1, 3, 5 modes, where transverse axes of oscillations are characteristic (Table 5).

Such a significant discrepancy in the experimental results (Table 7, Table 8, Table 9 and Table 10) during oscillations of the same plate, but from different sides, is explained by two technological factors: geometric deviations in the hole edges (Table 4, Figure 11), as well as deformation of the entire surface of the plate and giving it curvilinearity during industrial stamping. The decision was to average the experimental results *ω_ERA_*—average (Table 7) and *ω_EGA_*—average (Table 9), which made it possible to obtain sufficient accuracy of the results up to *δ_RA_* = 3.01% (Table 7) and up to *δ_GA_* = 4.64% (Table 9).

The deviation of the natural frequency of oscillations at the other modes (2, 4, 6–8) is in the range *δ_EG_* = 0.58–4.94% (Table 10).

The repetition of the deviation values of the natural frequency of oscillations, obtained experimentally and by means of FEM, confirms the assumptions made earlier.

The averaging of the natural frequency of oscillations of each side of the perforated surface *ω_EGA_* (Table 9) provided an increase in the accuracy of identification to *δ_GA_* = 0.24–4.64%, which is sufficient for further research or engineering design. It should be noted that increasing the edge area of holes with complex geometry, taking into account the technological consequences of their manufacture, leads to increased deviations *δ_GA_* (Table 9) in relation to round holes (Table 7).

The generalized results of the study of the natural frequency of oscillations of all surfaces are summarized in Table 11.

The final stage of these studies was the determination of the correction coefficients *K_p_* for the studied surfaces:

*K_p_*_1_ = (*ω_EGA_*/ω_ES_); *K*_p2_ = (*ω_ERA_*/*ω_ES_*), (10)

where *ω_EGA_*, *ω_ERA_*—averaged experimental values of the natural frequencies of oscillations of sieves with holes of complex geometry, and sieves with round holes, respectively; *ω_ES_*—the natural oscillation frequency of a solid surface.

The ranges of variation for the correction coefficients were determined as *K_p_*_1_ = 0.82–1.44 and *K_p_*_2_ = 0.84–1.08 (Table 12), which allows for the consideration of a complex set of holes, including those with complex geometry. The use of these obtained coefficients, *K_p_*, in the expressions (10) will enable the analytical determination of the natural frequency of oscillation for perforated surfaces with multiple holes in various modes.

Conducting experimental tests requires significant costs. A practical application can be the use of FEM to determine the correction factors *K_p_* for perforated surfaces with different holes:

*K_p_*_1_ = (*ω_FG_*/*ω_FS_*); *K_p_*_2_ = (*ω_FR_*/*ω_FS_*). (11)

Creating an adequate model in the Abaqus, for example, taking into account the specification of technological deviations (Table 4) and the use of comparative analysis will also allow to determine the correction factors necessary for analytical decisions *K_p_* for perforated surfaces with many holes of complex geometric shape.

The developed technique makes it possible to use the results obtained to substantiate the reliability parameters of perforated vibration plates with holes in the form of a five-petal epicycloid for certain conditions characteristic of the operation of separating calibration sieve machines. In addition, the developed methodology is based on certain stages: experimental, numerical, comparative analysis, and simplified analytical. This research algorithm has shown sufficient accuracy and adequacy and can be successfully implemented in the analysis of various designs of perforated vibration plates with different boundaries and initial conditions.

This work is the first stage in determining the reliability parameters of perforated plates with holes of complex geometry, the next stage of which will be the study of the occurrence and nature of the propagation of deformations in the form of cracks between the holes. The basic knowledge for the next stage is the obtained regularities of the natural frequency of oscillations of perforated plates obtained by simplified analytical, experimental, and numerical (FEM) methods. An important result is the possibility of using the obtained adequate numerical models (Abacus) for further study of the occurrence and propagation of cracks.

## 5. Conclusions

The methodology for investigating the natural oscillations of perforated surfaces has been developed and is based on the combined use of analytics, simulation FE-modeling, and experimental modal analysis.

The results of oscillation modeling of the perforated surface using the finite element method (Abaqus software) were compared with the measurements obtained from experimental modal analysis (Simcenter Testlab 2019.1 software). The research revealed differences in the results for the solid (non-perforated) surface, surfaces with round holes, and with holes of complex geometry (five-lobed epicycloids) at different modes. This led to the identification of correction coefficients that take into account the multitude of holes and their parameters.

The detected technological deviations in the manufacture of perforated surfaces and the way to account for them in FEM will significantly improve the accuracy of simulation modeling of oscillations.

The obtained dependencies of the natural frequencies of the investigated perforated surfaces take into account the presence of holes with complex geometry shapes and make it possible to make changes in the design to suppress vibration and change the nature of excitation in order to avoid entering into resonance.

This is of scientific and practical interest in the research, design, and improvement of the operational reliability of perforated surfaces with holes of complex geometry.

## Figures and Tables

**Table 1:** Coordinates of measurement point locations on the prototypes.

Perforated Surface Type	The Number of Points on the *x*-Axis	The Number of Points on the *y*-Axis	Distance between Points *l*_1_, mm	Distance between Points *l*_2_, mm
solid (without perforations)	10	7	69.5	41
sieve with HCG	11	10	65	30
sieve with basic round holes	10	7	69.5	41

**Table 2:** Characteristic of the test surfaces material.

Indicators	Values
Material and grade	still S235 JR
Yield strength, MPa	207
Tensile strength, MPa	345
Specific heat capacity of materials, J/(g °C)	0.455
The coefficient of thermal (linear) expansion, 10^−6^ m/(m °C)	17.44
Young’s modulus, GPa	210
Poisson’s ratio	0.3
Kirchhoff modulus, MPa	81.000
Density, g/cm^3^	7.847

**Table 3:** Technical characteristics of the accelerometer sensor.

Indicators	Values
Model number	352B10
Sensitivity (± 10%), mV/g	10
Measurement range, g pk	±500
Frequency range (± 5%), Hz	2–10.000
Broadband resolution (1 to 10,000 Hz), m/s^2^	0.03
Weight, g	0.7

**Table 4:** Geometric deviations of hole edges (*h* = 1 mm; R = 4 mm) during manufacturing.

Zone A				Zone B			
*l*_AX_, mm	Δ_AX_, %	*l*_AY_, mm	Δ_AY_, %	*l*_BX_, mm	Δ_BX_, %	*l*_BY_, mm	Δ_BY_, %
0.25	6.25	0.11	11	0.1	2.5	0.38	38

**Table 5:** Visual images of surface oscillations by various methods of study.

Mode	Number of Half-Waves (*x*,*y*)	Experiment	FEM
1	(1,1)	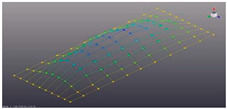	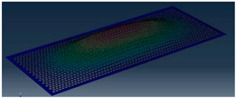
2	(1,2)	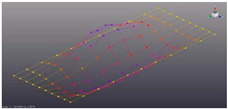	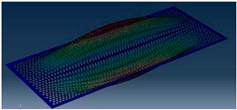
3	(2,1)	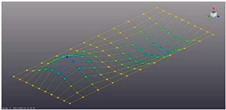	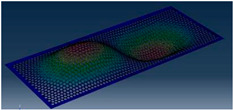
4	(2,2)	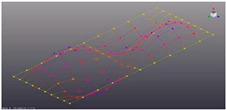	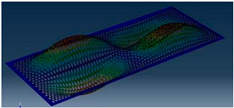
5	(3,1)	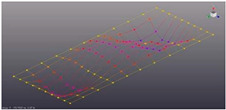	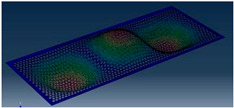
6	(3,2)	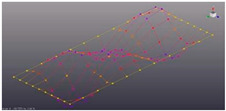	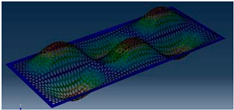
7	(4,2)	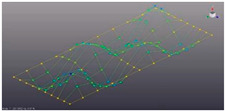	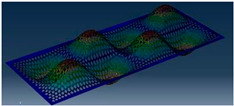
8	(5,2)	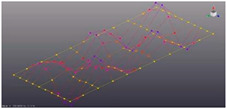	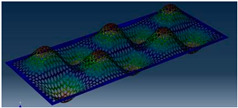

**Table 6:** The natural frequency of oscillations of a solid surface under various modes, which are obtained experimentally, analytically, and FEM (Hz).

Mode	Experiment *ω_ES_*	Analytical (Theoretical)		FEM *ω_FS_*	*δ_S_*, % *	
		α	*ω_TS_*		*δ_S_*_1_	*δ_S_*_2_
1	53.7	15.93	50.91	52.09	3.09	3.4
2	179.34	55.27	176.53	181.45	1.16	2.71
3	82.18	20.6	77.16	79.29	3.64	2.68
4	194.37	59.96	185.12	190.98	1.77	3.068
5	108.46	25.8	100.29	103.35	4.94	2.96
6	213.1	63.99	204.38	210.90	1.04	3.09
7	256.76	78.67	251.27	258.86	0.81	2.93
8	304.32	93.04	297.18	306.43	0.68	3.01

* δ_S__1_ = (*ω_FS_* − *ω_ES_*/*ω_FS_*) 100%, *δ_S_*_2_ = (*ω_FS_* − *ω_TS_*/*ω_FS_*) 100%.

**Table 7:** Natural frequency of oscillations of sieve with basic round holes with various modes, which are obtained experimentally and FEM (Hz).

Mode	Experiment			FEM *ω_FR_*	*δ_R_*, % *		
	*ω_ER_*_1_ (0°)	*ω_ER_*_2_ (180°)	*ω_ERA_*— Average		*δ_R_*_1_	*δ_R_*_2_	*δ_RA_*
1	59.24	52.92	56.08	55.64	6.47	4.89	0.79
2	156.86	148.78	152.82	152.45	2.89	2.41	0.24
3	90.22	78.66	84.44	81.97	10.06	4.04	3.01
4	175.3	166.01	170.66	167.39	4.73	0.82	1.95
5	124.72	110.53	117.625	115.78	7.72	4.53	1.59
6	202.12	192.71	197.42	200.89	0.61	4.07	1.73
7	226.88	216.62	221.75	225.78	0.49	4.06	1.78
8	262.68	248.53	255.61	252.56	4.01	1.60	1.21

* δ_R__1_ = (*ω_FR_* − *ω_ER_*_1_/*ω_FR_*) 100%, *δ_R_*_2_ = (*ω_FR_* − *ω_ER_*_2_/*ω_FR_*) 100%, *δ_RA_* = (*ω_FR_* − *ω_ERA_*/*ω_FR_*) 100%.

**Table 8:** Experimental values of the natural oscillation frequency of sieve with basic round holes under various modes and sides of the location (Hz).

Mode	*ω_ER_*_1_ (0°)	*ω_ER_*_2_ (180°)	*δ_ER_*, % *
1	65.24	48.92	10.67
2	154.86	149.78	5.15
3	96.22	68.66	12.81
4	174.3	169.01	5.29
5	116.72	94.53	11.37
6	202.12	190.71	4.65
7	220.88	216.62	4.52
8	262.68	248.53	5.39

* δ_ER_ = (*ω_ER_*_1_ − *ω_ER_*_2_/*ω_ER_*_1_) 100%.

**Table 9:** The natural frequency of oscillations of sieves with holes of complex geometry under various modes which were obtained experimentally and by FEM (Hz).

Mode	Experiment			FEM *ω_FG_*	*δ_G_*, % *		
	*ω_EG_*_1_ (0°)	*ω_EG_*_2_ (180°)	*ω_EGA_*—Average		*δ_G_*_1_	*δ_G_*_2_	*δ_GA_*
1	84.78	70.28	77.53	74.49	13.81	5.65	4.08
2	143.64	150.07	146.855	143.05	0.41	4.91	2.66
3	97.44	84.18	90.81	86.78	12.28	3.00	4.64
4	165.84	166.81	166.325	169.19	1.98	1.41	1.69
5	123.75	105.61	114.68	109.32	13.20	3.39	4.90
6	189.72	185.99	187.855	192.33	1.36	3.30	2.33
7	218.57	207.89	213.23	211.09	3.54	1.52	1.01
8	254.89	242.29	248.59	249.19	2.29	2.77	0.24

* δ_G__1_ = (*ω_FG_* − *ω_EG_*_1_/*ω_FG_*) 100%, *δ_G_*_2_ = (*ω_FG_* − *ω_EG_*_2_/*ω_FG_*) 100%, *δ_GA_* = (*ω_FG_* − *ω_EGA_*/*ω_FG_*) 100%.

**Table 10:** Experimental values of the natural frequency of oscillations of sieves with holes of complex geometry under various modes and sides of location (Hz).

Mode	*ω_EG_*_1_ (0°)	*ω_EG_*_2_ (180°)	*δ_EG_*, % *
1	84.78	70.28	17.10
2	143.64	150.07	4.48
3	97.44	84.18	13.61
4	165.84	166.81	0.58
5	123.75	105.61	14.66
6	189.72	185.99	1.97
7	218.57	207.89	4.89
8	254.89	242.29	4.94

* *δ_EG_* = (*ω_EG1_* − *ω_EG_*_2_/*ω_EG1_*) 100%.

**Table 11:** Comparison of frequency characteristics (Hz) of investigated surfaces obtained by different methods.

Mode	Solid Surface			Sieve with Holes of Complex Geometry			Sieve with Basic Round Holes		
	*ω_ES_*	*ω_FS_*	*δ_S_,* %	*ω_EGA_*	*ω_FG_*	*δ_G_,* %	*ω_ERA_*	*ω_FR_*	*δ_G_,* %
1	53.7	52.09	3.09	77.53	74.49	4.08	56.08	55.64	0.79
2	179.34	181.45	1.16	146.86	143.05	2.66	152.82	152.45	0.24
3	82.18	79.29	3.64	90.81	86.78	4.64	84.44	81.97	3.01
4	194.37	190.98	1.77	166.33	169.19	1.69	170.66	167.39	1.95
5	108.46	103.35	4.94	114.68	109.32	4.90	117.63	115.78	1.59
6	213.1	210.9	1.04	187.86	192.33	2.33	197.42	200.89	1.73
7	256.76	258.86	0.81	213.23	211.09	1.01	221.75	225.78	1.78
8	304.32	306.43	0.68	248.59	249.19	0.24	255.61	252.56	1.21

**Table 12:** Correction coefficients *K_p_* were obtained taking into account experimental data.

Mode	Experiment			*K_p_*	
	*ω_ES_*	*ω_EGA_*	*ω_ERA_*	*K_p_*_1_	*K_p_*_2_
1	53.7	77.53	56.08	1.44	1.04
2	179.34	146.85	152.82	0.82	0.85
3	82.18	90.81	84.44	1.11	1.03
4	194.37	166.33	170.65	0.86	0.88
5	108.46	114.68	117.63	1.06	1.08
6	213.1	187.86	197.42	0.88	0.93
7	256.76	213.23	221.75	0.83	0.86
8	304.32	248.59	255.61	0.82	0.84

## Data Availability

Data are available upon reasonable request to the authors.
